# 超高效液相色谱-串联质谱法检测动物源性食品中8种有机磷酸酯

**DOI:** 10.3724/SP.J.1123.2024.07010

**Published:** 2025-04-08

**Authors:** Yan TANG, Sheng WEN, Wencheng CAO, Xiao LIU, Chenglin LEI, Qingyun CHENG, Haichuan CHEN, Ling LIU, Xiaofang LIU, Yan ZHOU

**Affiliations:** 1.新疆大学生命科学与技术学院,新疆 乌鲁木齐 830017; 1. College of Life Sciences and Technology, Xinjiang University, Urumqi 830017, China; 2.国家卫生健康委员会食品安全风险评估与标准研制特色实验室,湖北省疾病预防控制中心,湖北 武汉 430079; 2. NHC Specialty Laboratory of Food Safety Risk Assessment and Standard Development, Hubei Provincial Center for Disease Control and Prevention, Wuhan 430079, China; 3.湖北工业大学生命科学与健康工程学院,湖北 武汉 430079; 3. School of Life and Health Sciences, Hubei University Of Technology, Wuhan 430079, China

**Keywords:** 有机磷酸酯, 动物源性食品, 超高效液相色谱-串联质谱, organophosphate esters (OPEs), animal-derived foods, ultra performance liquid chromatography-tandem mass spectrometry (UPLC-MS/MS)

## Abstract

采用乙腈作为提取溶剂,结合超声波辅助萃取和SPE柱进行前处理,建立了超高效液相色谱-串联质谱测定动物源性食品中8种有机磷酸酯(OPEs)的检测方法。准确称取0.5 g样品,用5 mL乙腈超声萃取;冷冻离心后取上清液,用HMR-Lipid SPE柱进行净化,待测分析物经C_18_ 色谱柱分离,在正离子模式下采集,2,2-二(氯甲基)-1,3-丙二醇双[双(2-氯乙基)磷酸酯]酯(V6)采用外标法定量,其他7种化合物(磷酸三乙酯(TEP)、磷酸三丙酯(TPrP)、磷酸三丁酯(TnBP)、磷酸三(2-氯乙基)酯(TCEP)、磷酸三(2-氯丙基)酯(TCIPP)、磷酸三(1,3-二氯-2-丙基)酯(TDCIPP)和磷酸三苯酯(TPHP))采用内标法定量。结果表明,该方法线性相关系数*r*^2^≥0.9900;各物质检出限为0.01~0.87 μg/kg,定量限为0.02~2.62 μg/kg;在2、20、100 μg/kg 3个水平下进行加标回收试验,回收率为80.5%~117.8%; RSD≤14.8%(*n*=6)。按此方法分析了12种动物源性食品样品(草鱼、鲈鱼、小龙虾、牛乳、奶粉、酸奶、猪肉、牛肉、鸡肉、鸭肉、鸡蛋和鸭蛋),其中化合物TnBP和TCIPP的检出率为100%, TEP、TCEP、TPHP和TDCIPP的检出率高于50%, TPrP和V6未检出。本方法前处理操作简单,分析速度快,回收率和精密度较好,适用于多种动物源性食品中OPEs的快速分析检测。

## Abstract (en)

Organophosphate esters (OPEs) are widely used as flame retardants in most regions, they adversely affect ecosystems and threaten human health. OPEs have attracted significant public attention because they are toxic and ubiquitously present in the environment. While China is among the world’s largest users and producers of OPEs, limited data on the exposure of animal-derived foods to OPEs exist; consequently, a method for quantifying OPEs in animal-derived food samples is needed.

In this study, a method was developed for the determination of eight OPEs, including triethyl phosphate (TEP), tripropyl phosphate (TPrP), tri-*n*-butyl phosphate (TnBP), tris(2-chloroethyl) phosphate (TCEP), tris(2-chloroisopropyl) phosphate (TCIPP), tris(1,3-dichloro-2-propyl) phosphate (TDCIPP), triphenyl phosphate (TPHP), and 2,2-bis(chloromethyl) trimethylene bis[bis(2-chloroethyl) phosphate] (V6), from twelve types of typical animal-derived foods by ultra performance liquid chromatography-tandem mass spectrometry (UPLC-MS/MS). The samples were purified using an HMR-Lipid SPE column. The effects of mobile phase A (water, 5 mmoL/L ammonium acetate aqueous solution, and 0.1% formic acid aqueous solution) and mobile phase B (methanol and acetonitrile), as well as the methanol/acetonitrile ratio on the separation and extraction efficiencies for the eight OPEs were investigated using one-way analysis. The results showed that optimal response values and peak shapes were obtained for the various compounds using 0.1% formic acid aqueous solution-acetonitrile system as the mobile phase. The following pretreatment procedure was used: A 0.5 g sample was accurately weighed and ultrasonically extracted with 5 mL of acetonitrile. The supernatant was collected after freezing and centrifugation, and cleaned-up was performed using an HMR-Lipid SPE column. The target analytes were analyzed using a Waters Acquity BEH C_18_ column (100 mm×2.1 mm, 1.7 μm) and ESI^+^ MS conditions. Compound V6 was quantified by the external standard method, with the other seven compounds quantified using the internal standard method. The method exhibited linearity with *r*^2^≥0.9900, limits of detection (LODs) of 0.01-0.87 μg/kg, and limits of quantification (LOQs) of 0.02-2.62 μg/kg for the various substances. Spiked recoveries of the eight OPEs at three levels (2, 20, and 100 μg/kg) were in the range of 80.5%-117.8% and RSDs ≤ 14.8% (*n*=6). Twelve animal-derived foods (grass carp, bass, *Procambarus clarkii*, milk, milk powder, yogurt, pork, beef, chicken, duck meet, egg, and duck egg) were analyzed using the developed method. Compounds TnBP and TCIPP were detected at rates of 100%, and TEP, TCEP, TPHP, and TDCIPP at rates greater than 50%, while TPrP and V6 were not detected. The method has a simple-to-operate pre-treatment step, analyzes rapidly with good recoveries and precisions, and is suitable for rapidly analyzing and detecting eight OPEs in a variety of animal-derived foods.

有机磷酸酯(organophosphate esters, OPEs)因具有良好的阻燃效果,被广泛用作阻燃剂、增塑剂、润滑剂、消泡剂和添加剂等,故OPEs成为溴化阻燃剂(brominated flame retardants, BFRs)的合适替代品之一^[1]^。人们日常生活中频繁接触的纺织品、电子产品、塑料制品、家具、建筑材料及食品包装中常常添加OPEs^[2]^。OPEs通常是以物理混合而非化学键合的方式添加到聚合物材料中,很容易通过挥发、浸出、磨损和溶解释放到大气中^[3]^。因此,在各种环境介质中,如降尘、水、沉积物、空气以及各类食物中已经检出OPEs。越来越多的研究表明,OPEs可能对人体健康带来危害^[4]^,不仅影响新陈代谢^[5]^,而且具有潜在的毒性,包括生殖毒性^[6]^、神经发育毒性^[7]^和甲状腺毒性^[8]^等。OPEs具有较高的亲脂性和半挥发性,具有一定的迁移能力,能够通过食物链进行富集^[9,10]^,目前欧盟和国内关于食品中OPEs的限值尚未有相关规定^[11]^,仅有少量研究调查了动物源性食品中OPEs的残留^[12,13]^。

目前,动物源性食品中OPEs的检测方法主要有气相色谱-质谱法(GC-MS)和液相色谱-质谱法(LC-MS)两类,使用GC-MS时,需要更长时间完成检测^[14,15]^,而相同待测物使用LC-MS则可以在更短时间内完成检测^[13,16,17]^。动物源性食品中OPEs前处理分为提取和纯化两步。常用超声辅助萃取(UAE)^[13,15,16,18,19]^、加速溶剂萃取(ASE)^[14]^或QuEChERS方法^[15,20,21]^使样品中的OPEs溶入有机溶剂中,后用固相萃取法(SPE)进行纯化,去除样品中的脂肪。但此类前处理方法多适用于脂肪含量低于20%的单一基质(如鸡蛋、鱼或牛奶),且前处理方法可进一步进行优化,从而提高方法的准确性与灵敏度。动物源性食品在我国居民日常膳食中占重要地位^[22]^,因此建立一种简单且能够准确测定多种含脂量较高的动物源性食品中OPEs的分析方法十分必要。

本研究采集了12类典型动物源性食品,先用超声辅助进行萃取,再用HMR-Lipid SPE柱进行净化,结合超高效液相色谱-串联质谱(UPLC-MS/MS)测定动物源性食品中8种OPEs,为动物源性食品中OPEs的检测提供理论依据。

## 1 实验部分

### 1.1 仪器与试剂

UPLC-TQ-X超高效液相色谱-串联质谱仪(美国Waters公司); VacElut 20固相萃取装置(美国Agilent公司); Multifuge X3R高速冷冻离心机(美国Fisher公司); D-78224型超声仪(德国Elma公司); M64全自动氮吹仪(莱伯泰科有限公司); DMT-2500多管旋涡混合仪(杭州米欧仪器有限公司); B-400均质仪(瑞士BUCHI公司); HMR-Lipid SPE柱(600 mg/6 mL,北京纳鸥科技有限公司)。

乙腈、乙酸铵(色谱纯,美国Fisher公司);甲醇(色谱纯,美国Merck公司);甲酸(德国CNW公司)、Milli-Q超纯水制备系统(美国Millipore公司)。

混合标准溶液(ES-5530, 10 μg/mL,包括磷酸三乙酯(TEP)、磷酸三丙酯(TPrP)、磷酸三丁酯(TnBP)、磷酸三(2-氯乙基)酯(TCEP)、磷酸三(2-氯丙基)酯(TCIPP)、磷酸三(1,3-二氯-2-丙基)酯(TDCIPP)、磷酸三苯酯(TPHP))和混合内标标准溶液(ES-5529, 10 μg/mL, TEP-D_15_、TPrP-D_21_、TnBP-D_27_、TCEP-D_12_、TCIPP-D_18_、TDCIPP-D_15_、TPHP-D_15_)购自加拿大Wellington公司; 2,2-二(氯甲基)-1,3-丙二醇双[双(2-氯乙基)磷酸酯]酯(V6, 50 μg/mL,挪威Chiron公司)。

### 1.2 实验方法

#### 1.2.1 标准溶液的配制

将混合标准溶液和标准原液V6用乙腈稀释为100 μg/L的标准中间液,混合内标标准溶液稀释为1000 μg/L的标准中间液。用乙腈溶液配制质量浓度为0.1、0.5、1.0、5.0、10.0、50.0 μg/L的系列标准工作液,每个质量浓度点均加入10 μL质量浓度为1000 μg/L的混合内标标准溶液。

#### 1.2.2 样品制备和处理

在湖北省城市点(荆州和十堰)和农村点(天门、枣阳、赤壁和红安)采集草鱼、鲈鱼、小龙虾、牛乳、奶粉、酸奶、猪肉、牛肉、鸡肉、鸭肉、鸡蛋和鸭蛋等12种动物源性食品样本共计72份,6个采样点同种类样本取相同重量混合,取可食部分充分混匀均质,制成12类湖北省代表性混样,置于-20 ℃冰箱储存。

准确称取0.5 g样品置于15 mL离心管中,依次加入100 μL 100 μg/L混合内标标准溶液和5 mL乙腈;在1500 r/min条件下涡旋5 min,经超声萃取30 min后,于-20 ℃冷冻30 min。然后在10000 r/min下离心5 min。取上清液采用HMR-Lipid SPE柱进行固相萃取处理,HMR-Lipid SPE柱先用5 mL乙腈-水(80∶20, v/v)进行活化。用5 mL甲醇-乙腈(70∶30, v/v)洗脱,收集洗脱液。随后在45 ℃条件下氮吹至近干,用1 mL乙腈复溶,涡旋1 min,超声2 min后,在10000 r/min下离心2 min后取上清液至进样瓶中待测。

#### 1.2.3 仪器条件

色谱柱:Waters Acquity BEH C_18_(100 mm×2.1 mm, 1.7 μm);捕集柱:Waters Isolator Column(50 mm×2.1 mm);流动相A: 0.1%(v/v)甲酸水;流动相B:乙腈;进样量:2 μL;流速:0.4 mL/min;柱温:40 ℃。梯度洗脱程序:0~0.3 min, 15%B; 0.3~5 min, 15%B~100%B; 5~6 min, 100%B; 6~9 min, 100%B~15%B。

离子源:电喷雾离子源(ESI);电离模式:正离子;扫描模式:多反应监测(MRM)模式;离子源温度:150 ℃;毛细管电压:0.5 kV;锥孔电压:30 V;溶剂化温度:550 ℃;脱溶剂气体流速:1000 L/h;锥孔气体流速:150 L/h。8种OPEs的其他质谱参数见表1。

**表1:** 8种目标物及其同位素内标的保留时间和质谱参数

Compound	CAS No.	*t*_R_/min	Parent ion (*m/z*)	Product ions (*m/z*)	DP/V	CEs/eV
Triethyl phosphate (TEP)	78-40-8	1.98	183.1	99.0^*^, 127.0	25	19, 12
Tripropyl phosphate (TPrP)	513-08-6	3.24	225.1	99.0^*^, 141.0	20	17, 10
Tri-*n*-butyl phosphate (TnBP)	126-73-8	4.30	267.1	99.0^*^, 155.0	30	17, 9
Tri(2-chloroethyl) phosphate (TCEP)	115-96-6	2.66	286.9	98.9^*^, 125.0	30	22, 16
Tris(2-chloroisopropyl) phosphate (TCIPP)	13674-84-5	3.44	327.0	99.0^*^, 175.0	30	19, 12
Triphenyl phosphate (TPHP)	115-86-6	4.20	327.1	215.0^*^, 152.0	30	26, 40
Tri(1,3-dichloro-2-propyl)phosphate (TDCIPP)	13674-87-8	4.03	430.8	99.0^*^, 320.9	30	24, 12
2,2-Bis(chloromethyl)trimethylenebis	38051-10-4	3.51	583.0	361.0^*^, 235.0	30	17, 30
[Bis(2-chloroethyl) phosphate] (V6)						
D_15_-Triethyl phosphate (TEP-D_15_)	135942-11-9	1.94	198.2	102.0^*^, 134.0	25	20, 12
D_21_-Tripropyl phosphate (TPrP-D_21_)	1219794-92-9	3.20	246.0	102.0^*^, 105.0	15	15, 10
D_27_-Tri-*n*-butyl phosphate (TnBP-D_27_)	61196-26-7	4.23	294.3	102.0^*^, 166.2	30	25, 25
D_12_-Tri(2-chloroethyl) phosphate (TCEP-D_12_)	1276500-47-0	2.65	299.1	130.0^*^, 102.0	30	16, 25
D_18_-Tris(2-chloroisopropyl) phosphate (TCIPP-D_18_)	1447569-78-9	3.41	345.1	102.0^*^, 183.0	25	22, 14
D_15_-Triphenyl phosphate (TPHP-D_15_)	1173020-30-8	4.16	342.2	223.0^*^, 262.1	30	25, 27
D_15_-Tri(1,3-dichloro-2-propyl)phosphate (TDCIPP-D_15_)	1447569-77-8	4.01	446.2	102.0^*^, 216.0	30	28, 16

*Quantitative ion. DP: declustering potential; CE: collision energy.

## 2 结果与讨论

### 2.1 质谱条件优化

OPEs目标物在正离子模式下进行离子化即可达到最优的灵敏度,故在电喷雾离子源正模式下进行全扫描,确定每一个目标物质的母离子,再进行子离子扫描,每个目标物质选取2对响应值高的特征离子对信息分别作为定量、定性离子对,再进一步对锥孔电压和碰撞能量进行优化,优化结果见表1。

### 2.2 流动相条件优化

本研究比较了流动相A(水、5 mmol/L乙酸铵水、0.1%(v/v)甲酸水)和流动相B(甲醇和乙腈)不同组合对分析结果的影响。结果显示,乙腈洗脱能力强于甲醇,能更快地将OPEs洗脱下来,进而减少整个方法的用时。且用乙腈洗脱时,各化合物响应值大于甲醇,峰形更好。5 mmol/L乙酸铵水-乙腈进行洗脱时无法实现TEP和TDCIPP的有效分离,两种目标化合物表现为重叠峰。以水-乙腈作为流动相时,TDCIPP重叠峰现象并未得到改善。当以0.1%(v/v)甲酸水-乙腈作为流动相时可实现两种物质的完全分离。在0.1%(v/v)甲酸水-乙腈流动相条件下,8种分析物的提取离子流色谱图见图1。

**图1:**
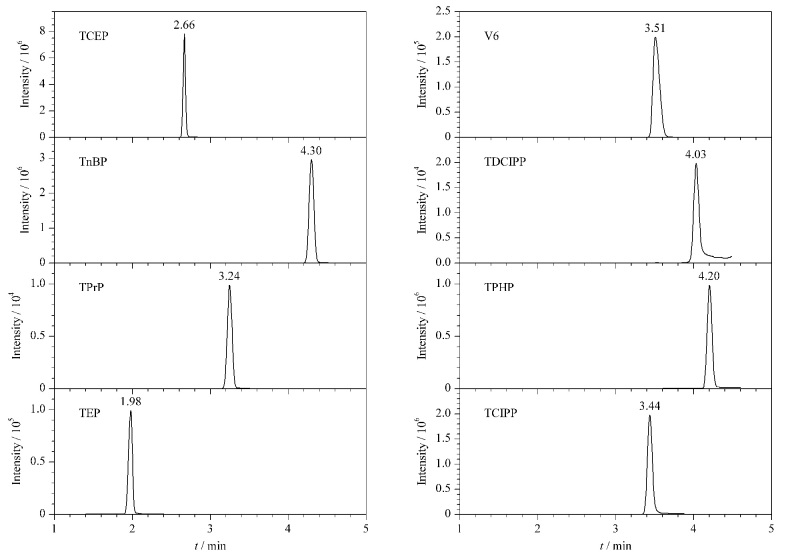
8种目标物的提取离子流色谱图

### 2.3 洗脱溶剂优化

由于HMR-Lipid SPE柱具有优异的亲水性,能够有效去除复杂基质中的脂质且不影响目标物的分析,故选择HMR-Lipid SPE柱进行样本净化。为了使回收效果更好,针对HMR柱净化方法做进一步优化。HMR柱为无机固相萃取柱,通常使用极性溶剂作为洗脱溶剂,实验选取甲醇和乙腈作为洗脱溶剂进行比较,发现除V6外,乙腈对其他目标物的洗脱能力均强于甲醇。为了提高8种目标物的回收率,设置了3种洗脱比例:甲醇∶乙腈=7∶3、1∶1、3∶7(v/v)。结果显示,V6的回收率得到了明显改善,甲醇-乙腈(70∶30, v/v)作为洗脱溶剂时,8种目标物的回收率达到最优(如图2所示)。

**图2:**
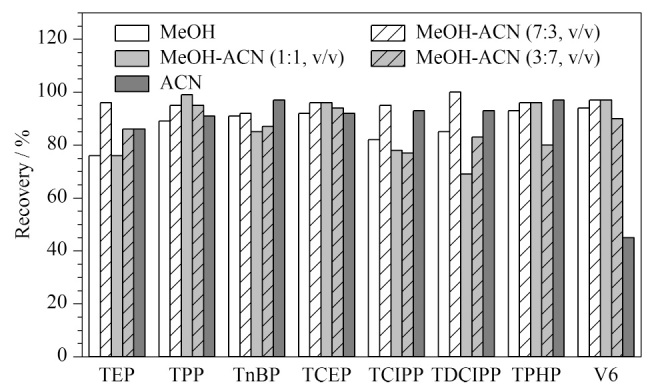
采用不同洗脱溶剂时8种目标物的回收率(*n*=3)

### 2.4 方法学验证

#### 2.4.1 标准曲线与检出限

按照仪器工作条件测定系列混合标准溶液,以离子对和保留时间定性,除化合物V6用外标法定量外,其他7种目标物均用内标法定量。8种OPEs的线性范围、线性方程和相关系数结果见表2。选择猪肉、鲈鱼、牛奶和鸡蛋4种典型动物源性食品基质分别进行方法学验证,根据空白标准偏差法评估LOD^[23]^,向猪肉、鲈鱼、鸡蛋和牛奶空白样品中添加0.1 μg/L混合标准溶液后进行分析,独立测试10次,计算标准偏差,LOD为样品空白值加4.65倍标准偏差,并以3倍LOD确定LOQ。结果如表2所示,8种OPEs在0.1~50 μg/kg范围内均具有良好的线性关系,*r*^2^为0.9900~0.9998, LOD为0.01~0.87 μg/kg, LOQ为0.02~2.62 μg/kg,说明本方法能够满足8种OPEs的测定需求。

**表2:** 目标物的线性方程、相关系数、检出限和定量限

Compound	Linear equation	*r*^2^		Pork						Bass						Milk						Egg			
				LOD/ (μg/kg)		LOQ/ (μg/kg)				LOD/ (μg/kg)		LOQ/ (μg/kg)				LOD/ (μg/kg)		LOQ/ (μg/kg)				LOD/ (μg/kg)		LOQ/ (μg/kg)	
TEP	*Y*=0.6593*X*+0.0272		0.9994		0.09		0.26				0.02		0.06				0.02		0.06				0.08		0.25
TPrP	*Y*=0.3096*X*-0.0038		0.9997		0.01		0.02				0.04		0.11				0.02		0.05				0.14		0.42
TnBP	*Y*=0.9581*X*+0.0663		0.9998		0.07		0.22				0.11		0.32				0.13		0.38				0.03		0.09
TCEP	*Y*=0.8667*X*+0.0590		0.9990		0.07		0.21				0.04		0.12				0.03		0.09				0.01		0.04
TCIPP	*Y*=0.9607*X*+0.9232		0.9900		0.44		1.31				0.55		1.64				0.47		1.42				0.87		2.62
TPHP	*Y*=4.0582*X*+0.0936		0.9994		0.07		0.21				0.03		0.09				0.02		0.07				0.06		0.17
TDCIPP	*Y*=1.5617*X*+0.1166		0.9992		0.02		0.06				0.02		0.05				0.02		0.05				0.01		0.03
V6	*y*=2734.09*x*+148.50		0.9991		0.04		0.11				0.02		0.06				0.03		0.08				0.03		0.08

*Y*: peak area ratio of target analyte to internal standard; *X*: mass concentration ratio of target analyte to internal standard; *y*: peak area; *x*: mass concentration, μg/L.

前处理方法中,使用HMR-Lipid柱和同位素内标法可以减少基质效应(ME)的影响,参考李艳明等^[24]^的方法,实验考察了8种OPEs在猪肉、鲈鱼、鸡蛋和牛奶中的基质效应,ME=(*a*_1_*-a*_2_)*/b*×100%,其中*a*_1_为OPEs在基质匹配标准溶液中的响应强度,*a*_2_为OPEs在空白基质溶液中的响应强度,*b*为OPEs在空白溶剂标准溶液中的响应强度。当ME>100%时,表示存在基质增强效应;当ME<100%时,表示存在基质抑制效应;当80%<ME<120%时,说明基质的影响较小。实验结果表明,8种OPEs的基质效应为82.7%~117.5%,大部分化合物受基质的影响较小,基质效应对8种OPEs的定量影响较小。

#### 2.4.2 精密度与回收率

在猪肉、鲈鱼、牛奶和鸡蛋样品中做3个水平6个平行的加标回收试验,计算各待测OPEs的平均回收率和相对标准偏差(RSD),结果见表3。8种OPEs的平均回收率为80.5%~117.8%,RSD为0.3%~14.8%,符合GB/T 27417-2017 《合格评定化学分析方法确认和验证指南》^[23]^中对回收率和精密度的要求,上述结果说明,本文所建立方法具有良好的回收率和精密度,且优于文献[13,14,17,20]中动物源性食品的加标回收率。

**表3:** 8种目标物在猪肉、鲈鱼、牛奶、鸡蛋中的加标回收率和精密度(*n*=6)

Compound	Added/ (μg/kg)	Pig			Bass			Milk			Egg	
		Recovery/%	RSD/%		Recovery/%	RSD/%		Recovery/%	RSD/%		Recovery/%	RSD/%
TEP	2	100.9	5.6		94.0	5.7		105.7	4.0		113.5	3.1
	20	98.4	1.4		82.2	0.3		105.8	0.8		100.4	1.4
	100	96.8	1.2		95.5	0.5		99.1	4.6		97.5	1.2
TPrP	2	93.4	3.8		86.8	3.0		97.4	7.4		98.1	9.3
	20	98.3	0.7		85.6	2.2		103.4	1.2		103.5	0.7
	100	92.4	1.2		95.1	3.7		100.9	2.7		95.1	1.2
TnBP	2	92.5	2.9		95.0	1.1		96.0	3.7		85.8	3.8
	20	109.8	1.9		93.9	3.6		115.3	1.2		113.4	1.9
	100	80.5	0.8		93.1	8.7		101.8	2.8		95.8	0.8
TCEP	2	96.3	12.1		99.1	4.2		87.1	3.1		82.4	6.8
	20	102.8	3.0		83.7	1.9		97.7	2.2		100.0	3.0
	100	101.1	1.0		97.8	2.2		94.0	4.0		96.3	1.0
TCIPP	2	106.8	3.5		106.5	0.7		110.2	1.3		87.9	5.2
	20	98.4	1.9		90.4	4.3		102.5	1.9		93.4	3.6
	100	101.5	2.4		98.4	4.1		96.4	3.8		98.4	4.5
TPHP	2	93.3	3.4		88.7	3.2		93.6	4.5		97.2	7.6
	20	90.0	2.6		94.6	4.0		102.3	3.0		109.3	2.6
	100	83.7	2.5		117.8	6.4		97.1	2.4		99.7	2.5
TDCIPP	2	96.1	10.7		85.3	5.3		95.5	3.0		81.9	8.0
	20	101.8	3.1		89.6	5.8		96.2	5.0		96.2	3.1
	100	103.4	2.0		101.6	2.0		91.8	4.4		88.1	2.0
V6	2	103.5	7.0		104.7	10.0		81.4	14.8		86.2	4.8
	20	86.2	2.7		93.2	4.5		85.0	5.6		97.0	2.7
	100	104.9	1.9		85.6	8.1		87.9	6.2		86.2	1.9

### 2.5 实际样品测定

应用本方法对湖北省6个采样点的12类动物源性食品混样进行检测。结果显示,在12类动物源性食品样品中,TnBP和TCIPP检出率为100%,含量分别为2.633~3.973 μg/kg和5.580~69.314 μg/kg; TEP、TCEP、TPHP和TDCIPP检出率分别为92%、92%、75%和50%,含量分别为0.238~101.638、0.042~2.638、0.040~0.518和0.139~0.199 μg/kg; TPrP和V6未检出(见表4)。

**表4:** 实际样品中8种目标物的含量(*n*=3)

Sample	TEP	TPrP	TnBP	TCEP	TCIPP	TPHP	TDCIPP	V6
Grass carp	0.721	<LOD	0.119	0.421	12.899	0.074	0.199	<LOD
Bass	2.564	<LOD	0.391	0.459	13.68	0.105	0.144	<LOD
*Procambarus clarkii*	0.797	<LOD	0.406	1.234	69.314	0.183	0.187	<LOD
Milk	0.238	<LOD	0.569	<LOD	37.243	0.04	0.185	<LOD
Milk powder	101.638	<LOD	0.932	2.633	7.252	0.518	0.139	<LOD
Yogurt	0.251	<LOD	0.288	0.064	47.359	0.064	0.149	<LOD
Pork	1.084	<LOD	0.252	0.171	11.15	0.121	<LOD	<LOD
Beef	0.661	<LOD	0.192	0.47	42.153	<LOD	<LOD	<LOD
Chicken	0.953	<LOD	0.128	0.462	5.58	0.133	<LOD	<LOD
Duck meet	1.162	<LOD	0.272	0.613	7.038	0.17	<LOD	<LOD
Egg	1.602	<LOD	0.269	0.472	23.829	<LOD	<LOD	<LOD
Duck egg	<LOD	<LOD	0.154	0.042	5.977	<LOD	<LOD	<LOD

本实验鱼类中化合物TEP、TnBP、TCEP和TCIPP均有检出,与文献中报道的研究结果一致^[14⇓⇓-17]^。鱼类中4种化合物检出含量(0.88~26.55 μg/kg)与广州市、北京市鱼类^[17]^中检出含量(5.94~33.7 μg/kg)相当,高于西班牙鱼类^[14]^中检出含量(0.25~3.35 μg/kg)。在湖北采集的小龙虾^[20]^样本中化合物TCIPP检出含量(9.72~13.67 μg/kg)低于本实验研究结果(69.314 μg/kg),化合物TEP、TCEP、TPHP和TnBP均高于本实验检出含量,OPEs的污染情况与采样地点有关。

## 3 结论

本研究采用UAE技术,利用HMR-Lipid柱进行净化,结合UPLC-MS/MS分析,建立了多种动物源性食品中8种OPEs同时检测的方法。本方法前处理操作简单,分析速度快,检出限低,回收率和精密度较好,适用范围广,满足动物源性食品中OPEs的痕量分析要求。本研究对湖北省动物源性食品中OPEs赋存情况进行调查分析,研究发现动物源性食品中OPEs普遍检出,个别种类含量较高,有机磷酸酯类阻燃剂的膳食摄入风险不明,本研究为动物源性食品中OPEs检测提供了方法学支持。但本研究目标化合物种类偏少,有机磷酸酯类阻燃剂的使用种类在持续增加,需进一步研究多种OPEs的高通量分析方法。
